# Social representations of the prevention of heterosexual transmission of HIV among young Africans from five countries, 1997-2014

**DOI:** 10.1371/journal.pone.0227878

**Published:** 2020-03-06

**Authors:** Kate Winskell, Robyn Singleton, Gaëlle Sabben, Georges Tiendrébéogo, Chris Obong’o, Fatim Louise Dia, Siphiwe Nkambule-Vilakati, Benjamin Mbakwem, Rob Stephenson

**Affiliations:** 1 Hubert Department of Global Health, Rollins School of Public Health, Emory University, Atlanta, Georgia, United States of America; 2 Institut de Recherche pour le Développement - Fondation Bouisson Bertrand / Montpellier-France; 3 School of Public Health, University of Memphis, Memphis, Tennessee, United States of America; 4 Super Buddies Club, Mbabane, Swaziland; 5 Community and Youth Development Initiatives, Owerri, Imo State, Nigeria; 6 Department of Health Behavior and Biological Sciences, University of Michigan School of Nursing, Ann Arbor, Michigan, United States of America; University of Pennsylvania, UNITED STATES

## Abstract

HIV prevention has evolved dramatically since the 1990s. The ABC trilogy (abstinence, be faithful, use a condom) has expanded to incorporate a range of biomedical prevention strategies, including voluntary medical male circumcision, pre- and post-exposure prophylaxis, and treatment-as-prevention, and to accommodate structural and combination prevention approaches. This study examines how young Africans from five epidemiologically and socio-culturally diverse countries (Swaziland, Kenya, Nigeria, Burkina Faso and Senegal) made sense of the evolving prevention of sexual transmission of HIV between 1997 and 2014. It uses a distinctive data source: 1,343 creative narratives submitted to HIV-themed scriptwriting competitions by young people aged 10–24. The study triangulates between analysis of quantifiable characteristics of the narratives, thematic qualitative analysis, and narrative-based approaches. Over time, HIV prevention themes become less prominent. Condoms are represented less often from 2008, though representations become more favourable. Biomedical prevention is all but absent through 2014. While prevention strategies may be described as effective in narratorial commentary, they are rarely depicted as preventing HIV, but are evoked instead in moralistic cautionary tales or represented as ineffective. Over time, an increasing proportion of protagonists are female. One in five narratives acknowledge structural drivers of HIV, but these are generally either disempowering or condemn characters for failing to prevent HIV in the face of often overwhelming structural challenges. In the context of combination prevention, there is a need to disseminate an empowering cultural narrative that models successful use of HIV prevention strategies despite structural constraints and avoids blaming and stigma.

## Introduction

In 2017, 1.8 million people were newly infected with HIV worldwide, a decline of 47% since incidence peaked at 3.4 million in 1996 [1]. The majority of these infections occurred in sub-Saharan Africa (SSA). Young Africans, particularly adolescent girls and young women (AGYW), continue to account for a high proportion of infections in SSA and there are fears of a resurgence in the epidemic if HIV incidence is not further reduced among African youth, as the largest ever generation of young people enters adolescence and adulthood [2]. There is, therefore, a pressing need to better understand the history and reception of HIV prevention efforts among young Africans and to draw implications for effective strategies for the future.

HIV prevention has evolved dramatically since the early days of the epidemic, not least in SSA. Abstinence, mutual monogamy and partner reduction, and the use of male condoms, along with testing, were mainstays of HIV prevention efforts in the 1990s [3]. Their popularization in the form of the ABC trilogy, inspired by the Bush Administration’s interpretation of Uganda’s successful efforts to curtail its epidemic in the 1990s, became enshrined in the contentious prevention policy of the US President’s Emergency Plan for AIDS Relief (PEPFAR) in 2003 [4].

Since the first HIV test became available in 1985, testing has played a role in HIV prevention not only as a diagnostic tool to allow behaviour change to prevent secondary transmission in the case of a positive result, but also–in combination with counselling–as a prevention strategy in its own right regardless of the test outcome, not least in the case of couple’s testing incorporating mutual disclosure [5]. More recently, testing is a precondition for or provides the gateway to a range of biomedical strategies to prevent sexual transmission of HIV. Post-exposure prophylaxis (PEP), the short-term use of antiretrovirals following exposure, has been acknowledged to reduce the risk of HIV infection since the early 1990s. Although PEP is acknowledged as an important intervention to prevent sexual transmission in SSA [6], its availability even in South Africa has been limited [7]. However, as a post-violence care intervention, it is currently a core component of the DREAMS (Determined, Resilient, Empowered, AIDS-free, Mentored and Safe) project to reduce HIV infections among AGYW in ten highly affected countries in SSA [8]. Between 2005 and 2007 Voluntary Medical Male Circumcision (VMMC) was shown to reduce HIV acquisition in men by approximately 60%; though coverage has varied by country, PEPFAR has reported supporting over 15 million VMMCs in southern and eastern Africa since 2007 [9]. A series of studies since 2010 have demonstrated protective effects of oral pre-exposure prophylaxis (PrEP) in preventing sexual transmission [10]. However, Kenya is the only country in SSA aggressively promoting PrEP [11].

In 2011, ART treatment was found to suppress viral load and thereby reduce transmission to sexual partners by 96% in stable couples, a phenomenon known as “treatment-as-prevention” (TasP) [12]. This resulted in WHO recommending in 2015 that all people living with HIV (PLHIV) initiate ART regardless of CD4 count (“universal test and treat”).

From 2004 [13], growing recognition of the feminization of the epidemic resulted in increased focus on its structural drivers. Structural approaches to HIV prevention “seek to change social, economic, political, or environmental factors determining HIV risk and vulnerability” [14], for example, through conditional cash transfers or efforts to reduce gender-based violence. Initially proposed by PEPFAR in its Second Five-Year Strategy in 2009, combination HIV prevention combines structural, behavioural, and biomedical interventions, often operating at mutually reinforcing levels of analysis in packages targeting specific key populations [15]. In 2014, in response to the gender disparity in HIV prevalence among adolescents (AGYW are 5–14 times more likely to be infected than their male peers), PEPFAR launched the DREAMS public/private partnership which delivers a core package of evidence-based approaches that address structural drivers of HIV risk, including poverty, gender inequality, sexual violence, and a lack of education, while also promoting behavioural and biomedical prevention strategies [8].

Remarkably little is known about how young Africans have made sense of these evolving HIV prevention technologies and strategies over time. It is also unclear how that sense-making differs across countries with diverse epidemiological and cultural environments. HIV-related stigma continues to have negative effects across the prevention and treatment continuum, creating a barrier to testing, disclosure, prevention strategies, treatment, adherence, and retention in care [16]. Comparing the social construction of HIV prevention among young Africans over time and across countries can help inform efforts to improve health outcomes and reduce stigma.

Coined by Moscovici in 1961 [17], social representation refers to the process whereby social knowledge is constructed and a shared system of meaning elaborated within and across social groups through processes of communication. The term also refers to the product of that process: the shared imagery, metaphors, values, and practices that allow us to make sense of, navigate, and position ourselves within the social world. Social representations are not static, but dynamic systems of social knowledge: they are created and recreated in everyday social interaction and spread through interpersonal and media communication. Narratives have been identified as a particularly valuable and underused data source for the study of social representations and sense-making [18, 19]. Often employed in studies of how new scientific ideas are integrated into lay thinking, social representations theory provides a particularly apt framework for a temporal study of representations of HIV.

Distinctive qualitative data from 47 countries, collected during a critical 18-year period (1997–2014) in the history of the epidemic, offer an opportunity to examine young Africans’ evolving social representations of HIV prevention. More than 150,000 young people from across SSA took part in HIV-themed scriptwriting contests held at 8 discreet time points between 1997 and 2014, contributing over 75,000 creative narratives from 47 countries. Although fictional stories are invented depictions of social fact, they are still culturally determined social facts in and of themselves and are a source of insight into how people make sense of the world, and how they communicate those understandings to others in their cultural community [20].

We analyzed young Africans’ social representations of HIV prevention in their creative narratives over a period of 18 years in five epidemiologically and socio-culturally diverse countries situated in West, East and Southern Africa: Senegal, Burkina Faso, Nigeria (South-East), Kenya, and Swaziland (now known as Eswatini). Our purpose was to identify needs and best practices and make recommendations for more effective messaging with a view to informing ongoing youth prevention efforts in response to an evolving epidemic.

## Methods

While Senegal is overwhelmingly Muslim, religious affiliation among the Burkinabe is more diverse, and the Igbo-speaking South-East Nigeria is, like Kenya and Swaziland, overwhelmingly Christian. Over the past eighteen years, the epidemic has evolved differently across the five countries as regards prevalence and ART coverage (Table 1).

**Table 1:** Adult HIV prevalence and ART coverage for the five study countries, 1997–2014 (Data from UNAIDS).

	Senegal	Burkina Faso	Nigeria	Kenya	Swaziland
**Adult HIV prevalence**					
**1997**	0.5%	3.2%	3.4%	11.1%	25.5%
**2005**	0.8%	1.5%	3.9%	7.4%	28.3%
**2008**	0.7%	1.2%	3.6%	6.4%	27.2%
**2014**	0.5%	0.9%	3.1%	5.7%	27.6%
**ART coverage**					
**2000**	0%	0%	0%	0%	0%
**2005**	1%	7%	3%	4%	6%
**2007**	17%	14%	5%	12%	14%
**2008**	21%	20%	8%	17%	19%
**2010**	28%	31%	11%	29%	33%
**2014**	39%	48%	23%	48%	60%

We analyzed de-identified narratives about HIV submitted to scriptwriting competitions by young people aged 10–24 at four discrete time points: 1997, 2005, 2008, and 2014. The competitions were coordinated internationally by the non-profit organization Global Dialogues (www.globaldialogues.org). Contest participants were invited to contribute a creative idea for a short film about HIV. The young participants in the Global Dialogues contests were mobilized by nongovernmental and community-based organizations and local, national, and international media across SSA. A leaflet, identical in all countries and available in several major languages, was used to provide young people up to the age of 24 with instructions on how to participate in the contest. From 1997 through 2011, six Scenarios from Africa contests invited participants to help others learn about HIV; in 2013 and 14, the contest, under the name Global Dialogues, was framed in global terms, included a broader array of themes (sexuality, violence against women, alcohol and drugs, in addition to HIV), and encouraged participants to speak out and participate in creating a better world. Scenarios were ineligible for inclusion in the study sample if they did not mention HIV. The shift in framing and elicitation did not result in noticeably different narratives on the theme of HIV from 2013 onwards. Although the competition was held eight times between 1997 and 2014, the current study addresses four discrete time points for which the data were qualitatively coded (1997, 2005, 2008, and 2014); data from 2013 are not addressed here for this reason.

We stratified our data into 12 categories by gender, urban/rural residence, and age of young author (10–14, 15–19, 20–24) and randomly selected 10 narratives from each of the 12 strata, oversampling locales if necessary to increase likelihood that 20 stories were selected for each age/gender stratum. In some countries, certain age/gender strata still contained fewer than 20 narratives, hence some country samples have fewer than the maximum 120 narratives. Our sampling procedures are described in detail elsewhere [21]. Data are only available for Senegal and Burkina Faso for 1997. Because of the historical importance of the years before 2005, when ART access increased dramatically, we include those data here. In light of the size and cultural diversity of the Nigerian population, only those narratives from the Igbo-speaking Southeast were sampled. These procedures yielded a sample of 1,343 narratives for the four time points (Table 2).

**Table 2:** Overall study sample (1997, 2005, 2008 and 2014).

Year	1997	2005	2008	2014	All
Burkina Faso	44	112	100	56	312
Kenya	N/A	88	25	116	229
Nigeria	N/A	120	93	88	301
Senegal	86	107	79	67	339
Swaziland	N/A	72	50	40	162
**All**	**130**	**499**	**347**	**367**	**1343**

### Data processing and analysis

Our methodological approach is situated at the intersection of grounded theory [22] and thematic narrative analysis [23] and triangulates between three primary analytical components: (1) analysis of quantifiable characteristics of the narratives (“quantitative attributes”); (2) thematic qualitative data analysis, focusing on thematically-related text segments and memoing for emergent themes; and (3) a narrative-based approach, focusing on plot summary and thematic keywords.

Our methods were developed to enable cross-national and temporal analysis and have three main advantages: (i) they ground the analysis in three distinct, though intersecting, dimensions of the data; (ii) allow triangulation; and (iii) facilitate the generation and validation of interpretive hypotheses.

We addressed the three primary analytical components as follows:

1. We quantified discrete components of each narrative, double-entering them into Qualtrics research software (Qualtrics, Provo, UT); in cases of discrepancy, consensus was reached via dialogue and/or adjudication by a third team member. Examples of these quantitative attributes of the narratives included whether a narrative includes a focus on prevention, infection, and/or life post-infection or whether it includes sexual transmission of HIV. Data were downloaded to Excel files; descriptive statistics were calculated in SAS software, Version 9.4 (SAS Institute Inc., Cary, NC).
2. Narratives (overwhelmingly handwritten) were transcribed into English or French and entered verbatim into MAXQDA 12 qualitative data analysis software (VERBI Software, 1989–2016), where they were labelled with reference to a codebook of 54 codes, including, for example, condom, abstinence, and fidelity/infidelity.
3. Each narrative was then summarized and independently double-coded with up to six out of 44 keywords, which included abstinence, condoms, and fidelity/infidelity. Discrepancies in coding were resolved through dialogue. The narrative summaries served as an aide-memoire and data-management tool, allowing us to easily identify common story arcs or to rapidly situate segments of data within the context of the overall plot or argument. The summaries also function in combination with thematic coding, allowing us to isolate both individual text segments related to a specific theme, for example, condoms, and those narratives in which condoms are a central theme.

Our qualitative analysis addresses both narratives coded with prevention-related keywords (3. above) and segments of other texts thematically coded with prevention themes (2. above). Existing thematic coding across a range of deductive (e.g. “poverty”) and inductive themes (e.g. “negative reactions to HIV diagnosis”) was complemented with fine-coding specific to themes related to the prevention of sexual transmission of HIV. Examples of fine codes include “barrier to condom: sexual violence” and “facilitator of abstinence: agency”.

This study, comprising the secondary analysis of existing data, was approved by Emory University Institutional Review Board. The narratives were provided for analysis by Global Dialogues (www.globaldialogues.org; info@globaldialogues.org). We cite them verbatim. Country names are abbreviated as follows: SZ–Swaziland; KY–Kenya; NG–Nigeria; BF–Burkina Faso; and SN–Senegal. Excerpts are identified by the country, contest year, gender, age, and geographic location of the author. The study’s limitations, related to its distinctive data source, are described at the end of the Discussion section.

## Results

Nineteen percent of narratives include a focus on prevention, in contrast to 69% on infection and 45% on post-infection. The proportion of narratives including a focus on prevention declines from a high of 24% in 2005 to a low of 11% in 2014; that including a focus on post-infection increases from 25% in 1997 to 56% in 2014; the proportion including a focus on infection is fairly consistent, but peaks at 76% in 2014) (Table 3). Narratives can include a focus on prevention, infection and post-infection simultaneously, for example, in cases of prevention within serodiscordant couples.

**Table 3:** Characteristics of narratives from 5 Countries (Senegal, Burkina Faso, Nigeria, Kenya, Swaziland) at 4 Time-Points (1997, 2005, 2008, 2014).

	TOTAL	1997	2005	2008	2014	Senegal	Burkina Faso	Nigeria	Kenya	Swaziland
Includes a focus on prevention (%)	**19**	19	24	22	11	15	21	19	24	19
Includes a focus on infection (%)	**69**	68	67	64	76	75	60	83	62	55
Includes a focus on post-infection (%)	**45**	25	36	54	56	32	42	45	56	62
Includes sexual transmission of HIV (%)	**60**	62	62	53	63	61	49	75	56	55
SEXUAL TRANSMISSION: Includes blame (%)	**53**	64	49	45	60	42	40	74	57	39
SEXUAL TRANSMISSION: Ends without hope (%)	**58**	70	62	41	64	59	47	70	60	43
Includes female protagonist(s) (%)	**67**	60	60	73	75	66	60	72	66	77
Includes male protagonist(s) (%)	**53**	73	51	56	44	59	64	43	48	41
Includes a female character acquiring HIV (%)	**72**	64	70	74	74	67	66	77	75	75
Includes a male character acquiring HIV (%)	**54**	72	53	52	49	56	60	48	53	50

Sexual transmission of HIV occurs in 60% of the 1,343 narratives, all of which include transmission between heterosexual partners (and, in five cases, also transmission between homosexual partners). Of the narratives featuring sexual transmission, 53% blame characters for becoming infected and 58% end without hope; these proportions are highest in 1997, but rebound in 2014 following lower levels in 2008. The proportion of narratives featuring sexual transmission ranges from 49% in Burkina Faso to 75% in Nigeria; among those featuring sexual transmission, the proportion including blame ranges from 39% in Swaziland to 74% in Nigeria and that ending without hope ranges from 43% in Swaziland to 74% in Nigeria.

A third of the narratives include a focus solely on heterosexual transmission, without depicting characters engaging in preventive strategies or living with HIV. However, over half of these “infection” narratives draw on prevention themes or contrast behavioural risk with preventive practices in character dialogue or narratorial commentary. For example, one narrative depicts Salif, who becomes infected through unprotected sex with sex workers; the narrative concludes, “Let’s be faithful and attentive because AIDS is wreaking havoc throughout the world. And, to avoid it, we need Abstinence, Faithfulness, and, if not, we need to have protected sex” (SN 1997, M 19 U). These narratives reflect “cautionary tales” [24], or cultural narratives that depict risk as preventable via self-monitoring and restraint and thus blame individuals for negative outcomes.

### Prevalence of prevention strategies

The prevalence of HIV prevention in the narratives differs by prevention strategy, by country, and over time. Of the ABC, condoms appear most prominently (mentioned in 33% of the 1,343 narratives and a central theme in 14%), followed by abstinence (mentioned in 19% and a central theme in 4%), and fidelity, infidelity and/or partner reduction (mentioned in 13% of the narratives and a central theme in 11%). Condoms referenced in the sample are almost exclusively male condoms; a female condom is mentioned in five narratives, in only one of which (a male-authored 2005 Kenyan narrative) it is used in a sexual encounter. Characters are tested for HIV in over half (n = 812, or 60%) of the 1,343 narratives, though this often serves to provide a turning point in the plot rather than being represented as a prevention strategy. Among the narratives in which testing occurs, 743 (92%) include positive tests, while 121 (15%) include negative tests; testing leads to characters taking action to prevent HIV transmission in 5% (n = 38) of these narratives.

Biomedical prevention methods are rarely mentioned or depicted in the narratives. Prior to 2014, male circumcision is represented as a traditional practice that is a mode of blood route transmission. VMMC for prevention purposes is ambiguously referenced in only one narrative, a 2014 text from Swaziland by a 19-year-old rural male author in which a male character, after having unprotected sex, questions the protection his circumcision offers. Only three narratives, all by 2014 male Kenyan authors, reference PEP in their narratives. These depict female-to-male transmission risk in which male characters access PEP to prevent transmission. PrEP and TasP are absent from the sample.

### Individual versus structural factors

While there are no representations of structural or combination prevention per se, representations differ in their emphasis on characters’ individual responsibility to protect themselves from HIV via behavioural strategies versus acknowledgement of the social and economic factors that increase vulnerability. Isolated narratives depict family members (NG 2008, M 18 R) or community organizations (BF 2008, M 22 R) providing economic support that allows female characters to avoid HIV risk associated with transactional sex, pointing to community-generated structural prevention efforts. However, the majority of representations of HIV prevention in the sample depict behavioural methods (i.e. abstinence, fidelity, condoms) for primary prevention.

Narratives emphasize individual responsibility for HIV prevention in 1 out of 3 narratives that include abstinence, fidelity/infidelity, partner reduction, or condoms. In these narratives, authors depict characters’ unwillingness or inability to put into practice these methods due to overwhelming lust, lack of perceived risk, or lack of knowledge about HIV infection. These narratives are most prominent in the Nigerian and least prominent in the Burkinabe sample.

One in 5 of the narratives highlight the social and economic vulnerabilities that place characters, overwhelmingly female, at risk of contracting HIV. For example, 1 in 3 narratives that include scenarios of condom use or non-use depict male characters refusing to wear condoms, threatening to withdraw economic support from female partners, or using sexual violence to obtain sex without a condom. Economic vulnerability, sexual coercion and violence, and male control of preventive practices (e.g. condom use) are among the primary obstacles to female characters successfully preventing HIV, and are most prominent in the 2014 sample. Narratives representing structural barriers are most common in the Swazi and Kenyan samples. While less blaming than narratives that solely emphasize individual responsibility for prevention, these narratives may reinforce a sense of tragedy and disempowerment in the face of HIV as narrators and characters lament the circumstances that facilitate HIV infection and inhibit prevention but rarely offer tangible ways to address them. One in 15 narratives, most prominently from Kenya and in the 2014 sample, acknowledge social and economic drivers of HIV risk while still emphasizing individuals’ responsibility to use HIV behavioural prevention strategies. Counter to the aims of structural and combination prevention, these narratives are particularly disempowering; for example, in an albeit extreme case, a narrative in which a girl is violently raped begins with the message to abstain from premarital sex (KY 2005, F 12 U).

### Gender

More narratives feature female protagonists over time, reaching 75% of narratives in 2014, compared to 44% with male protagonists. While more narratives depict male characters becoming HIV-positive in earlier years (72% in 1997 compared to 49% in 2014), the opposite is true for female characters (64% in 1997 vs. 74% in 2014).

The gender of protagonist differs by prevention strategy: the majority of the narratives in which condoms are a central theme depict male protagonists, while female protagonists predominate in narratives that include the abstinence theme. The overarching plotline and narrator commentary in the personal responsibility narratives reinforce moralistic messages that condemn active female sexuality, idealize female virginity, and connect HIV with immoral sexual behaviour; personal choices, particularly girls’ and women’s personal choices, are highlighted in these narratives as the driver of HIV risk.

### Effectiveness of prevention strategies

Condoms, abstinence, fidelity and, to a lesser extent, partner reduction are often framed as effective HIV prevention methods via narrator commentary but are rarely depicted within a plotline as successfully preventing HIV transmission. For example, although narrator commentary cites abstinence as an effective HIV prevention method (specifically for unmarried youth) in 1 in 4 narratives that include the abstinence theme, fewer than half of these depict characters successfully protecting themselves via abstinence. For male characters, the most common barriers to abstinence are peer pressure and overwhelming sexual desire. Female characters, while facing these individual-level barriers as well, are also depicted as pressured, coerced, or forced into sexual intercourse by male partners, 1 in 5 of whom are in an economically advantageous position.

Condoms are depicted as not used (i.e. “unprotected sex”) approximately four times more often than they are used. When the young authors depict condom use, half of the time they portray characters becoming infected with HIV despite having used condoms. While these narratives occur in all countries, they are particularly prominent in the Nigerian sample where condom *use* results in HIV transmission four times more often than it prevents HIV transmission. As with representations of abstinence, the most common barriers to condom use for male characters are individual-level (specifically, lack of perceived vulnerability to HIV infection or loss of control due to lust), whereas female characters are most often challenged by partner refusal that is exacerbated by economic vulnerability, or by sexual violence.

Fidelity is frequently represented as ineffective; for example, 3 out of 4 characters practicing fidelity become infected. In these cases, infections occur primarily as a result of a partner’s inability or refusal to remain sexually exclusive or due to infection via blood. Male characters are depicted as facing similar challenges to fidelity as to abstinence (e.g. lack of perceived vulnerability to HIV, sexual desire for and access to female partners). Female infidelity is most often linked to the economic incentive of having multiple partners.

In all three cases in which PEP is referenced, it is highlighted as an effective prevention strategy in the case of risky sex or female-on-male sexual violence. Only male characters, two out of three of whom are depicted as sexually assaulted by women, use PEP; despite the much higher proportion of male-on-female sexual violence in the 2014 sample (57 female victims versus 7 male victims), no narratives mention PEP in the case of rape of female characters.

### Temporal characteristics

Representations of HIV prevention in the narratives evolve over time. Where the 1997 sample focuses on the basic facts of transmission and prevention, the 2005 sample is distinctive for providing contextualized representations of successful prevention. The 2008 sample focuses more on testing as a gateway to treatment, while the 2014 sample sees a decline in prevention and increased preoccupation with the circumstances of infection.

Mentions of condoms and fidelity decline over time, while abstinence is more consistently present. The treatment of abstinence as a central theme peaks in 2005. In comparison with other years, the 2005 sample is distinctive in including a higher proportion of representations of successful abstinence, in which the combination of individual agency, self-control and supportive peers, partners and parents contribute to characters being able to choose to delay or avoid sex. 2014 narratives, in contrast, place less emphasis on the social facilitators of abstinence and depict greater challenges in terms of coercive relationships, while still underscoring the importance of individual agency in the choice to remain abstinent despite an unfavourable environment.

In 1997 (when data come only from Senegal and Burkina Faso), representations of condom use are rare but condoms are *mentioned* in over half of the narratives, often in combination with abstinence and partner reduction as the young authors underscore the need to prevent HIV in the absence of treatment. The use of humour to promote condom use, distinctive in the 2005 sample, disappears in subsequent years. In addition, the 2005 sample includes more characters, primarily female, negotiating condom use and refusing to have sex without one. Over time, condoms are mentioned and represented less often but more favourably. Representations of condoms as ineffective, either via descriptions of condoms being misused, breaking or simply failing to prevent HIV transmission, which recur in 2005 narratives from Nigeria in particular, become virtually absent in 2014. In 2008 and 2014, a handful of narratives (n = 6) depict the use of condoms to prevent transmission within serodiscordant couples.

### Cross-national characteristics

The prominence of narratives featuring individual responsibility and/or structural vulnerability differs by country. While blame and moralizing around HIV prevention that emphasize individual responsibility are found in all countries, they are most common in the Nigerian and, to a lesser extent, the Kenyan sample. Reference to structural vulnerability, particularly in Swazi narratives, tempers the blame authors ascribe to characters for their inability to prevent HIV.

#### Nigeria

The Nigerian sample is distinctive in the extent to which HIV infection is associated with “immoral” behaviours (pre- and extra-marital sex). Attempts to circumvent these moral ideals via condoms often fail, above all in the 2005 sample. One narrative format characteristic of the Nigerian sample contrasts “good girl” and “bad girl” archetypes to condemn sexual promiscuity and exalt abstinence: for example, Queenie is depicted as spoiled, sexually promiscuous and materialistic and ultimately dies a humiliating death from HIV, while poor and humble Munachi refuses to engage in transactional sex and rejects sexual advances as she works studiously through school, eventually marrying “one of the decent boys” (NG 2014, M 15 R). Positive and/or negative peer influence plays a prominent role in roughly half of the narratives in which abstinence or condoms are central themes. In contrast to other country samples, female characters in Nigerian narratives are represented as having multiple partners as often as male characters; they are primarily motivated by a desire for wealth and gifts, rather than forced by poverty.

#### Kenya

The Kenyan sample includes the highest proportion of representations of poverty, which serves as a barrier to HIV prevention, especially abstinence. It also includes the lowest proportion of narratives depicting an enabling environment for behavioural prevention, in the form of peer, partner, family or social support. Despite acknowledgement of the role played by poverty, stigma and lack of support in inhibiting HIV prevention, Kenyan narratives often emphasize characters’ personal responsibility to protect themselves from HIV. Although the Kenyan sample contains the highest proportion of narratives with prevention themes and hopeful endings that depict the challenges and obstacles characters face when attempting to enact prevention strategies, they often incorporate moralistic commentary that criticizes characters for failing to control themselves. The Kenyan sample is the only one to include reference to ARV-based biomedical prevention, namely PEP following female-to-male transmission risk.

#### Senegal

The Senegalese sample includes the lowest proportion of narratives that include a focus on prevention. Although representations of successful prevention are particularly rare in the Senegalese sample, narratives depict a supportive social environment and relatively few economic barriers to HIV prevention. The Senegalese sample includes the second highest proportion (after Burkina Faso) of references to the effectiveness of condoms and narratives depicting peer, partner and medical provider support for condom use, although representations of condom use are rare, as in other country samples. However, condoms are often associated with particular groups represented as being at high risk of HIV, specifically sex workers, migrants, individuals with multiple partners and foreigners. Senegalese narratives are distinctive in depicting male travel and labor-driven migration as a primary driver of infidelity by increasing wealth and subsequent access to sexual partners. For example, one narrative describes a young married man who travels to Europe for work. Upon earning money, “he forgot his wife…and began to throw himself into the arms of the Europeans. One day he had sex with a prostitute who was HIV positive” and subsequently infects his wife and, through mother-to-child transmission, their unborn child (SN 2008, M 22 U).

#### Swaziland

Swazi narratives are distinctive in their emphasis on the social context that contributes to HIV risk, particularly the gendered vulnerability experienced by female characters due to poverty, sexual pressure, coercion or violence, and resistance of male characters (often older and providers of economic support, affection and social status) to prevention methods. They depict the challenges of prevention, for example, peer pressure, poverty and gender inequality, but also–particularly in 2005 –empowered female characters successfully navigating these challenges and abstaining. In one narrative, for example, Gertrude refuses to have sex with Michael despite his attempts to pressure her, including via financial support (SZ 2005, F 17 R). The majority of Swazi texts that include the theme of condoms focus on dynamics within couples rather than casual sexual encounters or relationships with sex workers.

The Swazi sample includes the highest proportion of narratives depicting characters navigating life with HIV, without engaging with the circumstances of infection. As a result, it includes fewer depictions of testing than other countries as more narratives begin with characters knowing their status. Within the texts that include testing, Swazi authors include the highest proportion both of pre- and post-test counselling and of characters taking steps to live healthy lives with HIV and/or prevent further transmission following diagnosis. Despite an emphasis on living with HIV, Swazi authors rarely incorporate secondary prevention into their narratives. As a result there is a relatively low proportion of prevention narratives in the Swazi sample. Lastly, the Swaziland sample includes the only narrative that references VMMC, although the character questions its effectiveness for preventing HIV (SZ 2014, M 19 R).

#### Burkina Faso

Alongside the Swazi narratives, those from Burkina Faso are among the least moralistic in relation to sexual transmission and HIV prevention methods, with only 40% assigning blame to individuals for HIV transmission. Condoms are more prominent in the Burkina sample than in any other sample. Here, condoms, testing, partner reduction and secondary abstinence are presented as a menu of prevention options available to characters when navigating HIV risk. Importantly, these behavioural strategies are not restricted to specific “at-risk” populations nor morally condemned as the failure to uphold a moral ideal, but rather represented as pragmatic solutions to sexual risk. The Burkina sample includes more than twice the proportion of negative tests as other country samples. While acknowledging the role of poverty and economic barriers in sexual risk, particularly for female characters, Burkinabe narratives include the highest proportion of supportive social environments for HIV prevention.

### Empowering narratives

A small minority of narratives depict the successful prevention of HIV in ways that do not perpetuate HIV stigma or reinforce a sense of hopelessness or disempowerment. Narratives with successful prevention feature female empowerment and male respect for female characters’ desires. For example, while fewer than 1 in 25 narratives that include condoms depict successful condom use that prevents HIV, these positive representations are facilitated by supportive peers, community leaders and, for female characters, male partners who accede to their requests for protection. In one narrative, Assetou is propositioned by and initially refuses Germain. Her friends tell her about the risks of unprotected sex and urge her to use a condom. Assetou and Germain decide to have sex with a condom (BF 2008, F 21 U). Underlying these positive representations of HIV prevention lies a lack of stigmatization of sexuality in general and youth sexuality in particular. Sex outside marriage in these scenarios is not a deviant act worthy of punishment via HIV infection, but rather a common experience that carries risk that can be navigated with a menu of prevention strategies, deployed depending on the circumstances. Prevention methods therefore represent opportunities to navigate sexual risk rather than being indicative of personal failure. Gender and economic disparities are acknowledged but counteracted by supportive social environments that make it easier for female characters to exercise agency. These scenarios feature most frequently in the Burkina and Swazi samples.

## Discussion

In terms of prevention strategies, the most notable findings from our analysis are the decline in prominence of HIV prevention themes, the reduction in thematic prevalence of condoms, and the virtual absence of any reference to biomedical prevention strategies. Of particular relevance to representations of the social and structural drivers of HIV are the growing proportion of female protagonists, the predominance of moralistic cautionary tales, and the distinctive cross-national differences.

### Prevention strategies

The decline in prominence of HIV prevention themes intersects with increased access to life-extending ART (particularly evident in the 2008 sample) and a resurgence in moralistic preoccupation with the circumstances of infection in 2014 (following lowest levels of blame and hopelessness in 2008). It makes sense that young Africans’ narratives about HIV in the mid-to-late 2000s would include ART, following acceleration of access in the mid-decade. However, it is important to ensure that prevention remains a prominent dimension of young Africans’ thinking about HIV.

While it is encouraging that virulently moralistic condemnations of condoms as ineffective in the 2005 sample–which we associated with PEPFAR priorities and the prominence of the faith-based response [25]–have receded, their declining prominence in the narratives is of concern. Promotion and provision of male and female condoms is part of the DREAMS core package, launched in 2014, along with PrEP and testing [8]; both Kenya and Eswatini are part of the DREAMS partnership. It is, however, essential to ensure that condoms remain a central component of meaning-making around HIV prevention for young Africans in general, not just those at particular risk in higher-prevalence countries.

Warning against dangerous complacency, a 2018 Lancet Commission report [2] argues that the dominance of HIV treatment and biomedical approaches has resulted in the neglect and underfunding of primary prevention, along with efforts to address HIV-related stigma and other social and structural drivers. The shift of emphasis we observed in the narratives may reflect a declining prioritization of general youth prevention in the context of the increasing biomedicalization of the epidemic, as prevention has become less a matter for the general population and more focused on key populations and more clinic-based [26]. While mass media and community-level communication strategies were critical to the HIV response in the early days of the epidemic, they may be less prominent in light of more targeted approaches (summed up in UNAIDS “Know your epidemic, know your response” priority) [27]. In neglecting the general population for prevention messaging in favour of key populations, we exclude both those who are not easily reached by more targeted communication strategies and those who can help catalyze and sustain a supportive and enabling environment. This applies as much to biomedical prevention interventions [28] as to condom promotion.

While evidence supporting the major biomedical prevention strategies was in place before the 2014 scriptwriting contest–VMMC (2005–7), PEP (starting from the early 1990s), PrEP (from 2010) and TasP (2011)–these are virtually absent from the young authors’ narratives. This may reflect limited relevance, availability and/or awareness (depending on the strategy and the country). The countries in our study had baseline levels of male circumcision over 90% with the exception of Western Kenya and of Swaziland (which had a male circumcision prevalence rate of 8.2% in 2006–7 [29] but where VMMCs have increased substantially since then [9]). As only a proportion of our Kenyan texts are from Western Kenya, it makes sense that VMMC should feature most prominently in the Swazi sample, though it is disappointing that it is mentioned in only one 2014 text. We have been unable to locate data on PEP coverage in SSA; Ncube et al. [7] note that its availability is limited even in South Africa. PrEPWatch [30] reports the number of PrEP users per country. While Senegal is reported as having no users, as of June 2019, Kenya has the largest program in SSA and is reported to be the only country in SSA that is aggressively promoting PrEP use [11].

In summary, while limited relevance or availability accounts for silence around VMMC, PEP and PrEP in most countries in 2014, the silence surrounding TasP in all countries is of concern, given its universal relevance and its potential to benefit stigma reduction, testing, and treatment initiation and adherence. This silence is in keeping with qualitative studies conducted in 2013 in urban communities in South Africa and Zambia [31] and in 2015 among rural HIV-positive and HIV-negative South African men [32], which found no awareness of TasP. Further research is needed to determine to what extent this situation has changed in the intervening period and the extent to which it is attributable to policymakers’ and others’ reluctance to explicitly promote TasP for fear that it may lead to behavioural disinhibition and a rebound in unsafe sexual practices [32]. Continued low awareness of TasP would potentially be depriving SSA of a valuable tool in HIV prevention, treatment and stigma-reduction.

### Social and structural drivers

The growing feminization of the epidemic is reflected in an increasing proportion of narratives with female protagonists. Whether the young participants in the scriptwriting competitions observed this phenomenon directly in their communities or indirectly via media representations reflecting epidemiological understandings, they are increasingly propagating and reinforcing a social representation of a feminized epidemic in their narratives. The young authors understandably struggled to reconcile acknowledgement of structural drivers of HIV with individual preventive behaviours, creating disempowering narratives of vulnerability or condemning characters for failing to prevent HIV in the face of often overwhelming structural challenges. In the context of combination prevention, an empowering cultural narrative that models prevention despite structural constraints and avoids stigma or blame is increasingly important. Such a narrative should incorporate biomedical prevention strategies and help build demand for them: as Ahmed et al. [33] suggest, for example, it is critical that PrEP roll-out use a gain-framed and empowering approach.

Social representations can be viewed as a form of social norms in symbolic form. They interact with cultural narratives in a mutually constitutive way (manuscript under review). The data–in Nigeria, in particular–point to a well-established cultural narrative of HIV prevention that takes the form of a moralistic, cautionary tale and that, our 18 years of data suggest, has considerable staying power. Cautionary tales as conceptualized by Moore [24] place primary emphasis on personal responsibility for risk, such that risk becomes a moralizing discourse and allow for individual blame for outcomes. In this way, cautionary tales support the maintenance of stigma by associating HIV infection with immoral behaviours and populations while constructing the disease as preventable via individual conduct [34]. Our analysis of a broader sample (manuscript under review) suggest that the hope-promoting and stigma-reducing influence of the advent of ART access on symbolic representations in the narratives (evident in the 2008 data in particular) may have partially run its course by 2011/2013. The three countries where DHS temporal data on stigma is available show a downturn in accepting attitudes towards people living with HIV among 15–24 year-olds (for Senegal from 2010, for Nigeria from 2013, and for Kenya from 2014) [35]. This points to the need to redouble stigma reduction efforts among youth and to attend to the ways in which HIV communication may be fuelling stigma.

A cultural narrative compatible with combination prevention is, by virtue of its complexity, unlikely to be as compelling as cautionary tales of infection, that seek to capitalize on the deterrent effect of a loss-framed message [36]. The dominant cultural narrative may be resistant to accommodating new information and approaches that lend themselves to less melodramatic and moralistic storylines. Moralistic dimensions of the narratives, particularly in relation to gender and sexuality, reflect power-laden positions and appear least susceptible to change, even in the context of dramatic increase in access to ART. Nonetheless, cross-national differences and the existence of empowering narratives in our sample indicate the potential for alternative framings.

The cross-national differences in the narratives, particularly as they relate to sexual morality, blame and hope, are salient. Our analyses extend temporally our cross-national study of symbolic stigma in the 2005 sample [37], with a specific focus on HIV prevention. They demonstrate the consistency of cross-national differences over time, with representations in the Nigerian sample remaining the most stigmatizing, a finding that is statistically significant across a wider range of time points (manuscript under review). Our findings point to the need for HIV communication in Nigeria to address the stigmatizing link between HIV and sexual behaviours represented as immoral. This can potentially be done by elevating empowering narratives that depict a supportive social environment for HIV prevention, possibly drawing on the pragmatic menu of prevention options present in Burkinabe narratives. Young people in Nigeria also need to be encouraged to critically reflect upon the social and economic drivers of women and girls’ vulnerability to HIV, including sexual violence. Kenyan narratives continue to convey distinctively mixed messages, combining blame with acknowledgement of structural barriers. This characteristic, combined with the absence of representations of supportive social environments, points to the need to promote empathy with young Kenyans who are vulnerable to HIV and to disseminate empowering narratives that emphasize the agency of those at risk. There is also a need to go beyond messages of personal responsibility for infection and embrace messages about the collective responsibility to create a supportive social environment for HIV and violence prevention and response. Narratives from lowest prevalence Senegal remain ‘othering’ in their focus on ‘high risk’ groups and migration as a primary risk factor, suggesting a priority need to promote greater awareness of risk perception among Senegalese youth. Narratives from highest prevalence Swaziland, in contrast, continue to reflect the social proximity of the epidemic and awareness of structural factors, while the Burkina Faso sample is characterized by a non-stigmatizing pragmatism. While narratives from Swaziland and Burkina Faso have distinctive strengths from which other countries can learn, it is important to avoid complacency and remain vigilant in relation to stigma. In Swaziland in particular, our data point to a need for greater focus on secondary prevention, while in Burkina Faso, they point to a need to raise awareness of sexual violence, which is largely absent from the narratives.

Across all countries, we recommend increased focus on positively-framed prevention narratives–potentially drawing on examples in the 2005 and Burkina Faso samples–featuring empowered characters (male and female) creatively navigating circumstantial (and, where appropriate, structural) challenges to protect their health and achieve desired outcomes. These narratives can provide alternative role models for young people, cultivate skills development, boost their belief in themselves and their capacities, and align with combination prevention. While these narratives may lack the melodrama of the cautionary tale, their positive emotion can be leveraged in health education and communication materials and curricula to model and build skills around the successful application of prevention strategies.

### Limitations

This study has limitations related to its distinctive data source. Data for 1997 are available from only two countries, while those from Nigeria come only from the Igbo-speaking South-East. As contest participants self-select, the data are not representative of the youth populations; participants in the scriptwriting competitions may be better educated and more knowledgeable and motivated about HIV than the general youth population. As a product of the same contest mechanism, however, these biases are likely to be consistent across the five countries hence the country samples, though not representative, are comparable for our purposes. We have little demographic information about individual participants other than their gender, age, country of origin, and type of place of residence. In light of the circumstances in which the texts were written, it is impossible to know which depict lived versus imagined experience. The data are embedded within cultural norms of performance, discourse and persuasion [38], which may be informed by rhetorical considerations specific to the scriptwriting competition, reflecting the young authors’ motivation, for example, to tell what they consider to be a good story and thereby win the contest. The programmatic context of the scriptwriting competitions, including the shift in elicitation and broadening of themes from 2013, may have influenced the consistency of the data in ways that it is impossible for us to gauge. Despite their limitations and in the absence of comparable temporal and cross-national data, the narratives provide rare and distinctive insight into evolving sense-making about HIV prevention among a presumed general population of young Africans from five countries and across a time period of shifting strategies and programmatic priorities in efforts to prevent the sexual transmission of HIV.

## Conclusion

While our distinctive data have limitations, they provide a rare temporal and cross-cultural overview of young Africans’ symbolic representations of HIV prevention, identify country-specific needs, and point to strategies for future communication programming. The narratives suggest that, despite the temporary stigma-reducing dividend of increased access to ART, youth sense-making around HIV prevention continues to be dominated by cautionary tales and by moralistic messaging that fails to engage with the factors that constrain individual agency and that fosters stigma and victim-blaming. There is a risk that as mass media and community-level HIV communication recedes, this stigmatizing “prevention” narrative focused on the circumstances of infection increasingly fills the void. Our findings suggest both intervention needs and, particularly through the empowering narratives in the sample, the potential for more positive framings. They point to the necessity of ensuring that traditional prevention strategies–like condoms–*remain*, and new biomedical prevention strategies increasingly *become*, a central component of young Africans’ thinking about HIV. They suggest the pressing need to disseminate empowering narratives compatible with combination prevention, narratives that avoid blaming and stigma and model successful use of HIV prevention strategies, including biomedical prevention and TasP, despite structural constraints. Our findings further point to the importance of tracking social representations of HIV–and related cultural narratives–over time and place in efforts to better understand the intersections between youth HIV prevention and stigma and how they can best be addressed in communication practice.
